# The Appropriate Use and Care of Peripheral Intravenous Cannulas: A Quality Improvement Project

**DOI:** 10.7759/cureus.76954

**Published:** 2025-01-05

**Authors:** Shoon Lae Aung, Aditya Sengupta, Nwe Ni Win, Jeyanthy Rajkanna, Samson O Oyibo

**Affiliations:** 1 General Medicine, Peterborough City Hospital, Peterborough, GBR; 2 Internal Medicine, Peterborough City Hospital, Peterborough, GBR; 3 Diabetes and Endocrinology, Peterborough City Hospital, Peterborough, GBR

**Keywords:** care bundle, clincal audit, intravenous cannulation, peripheral iv cannulation, quality improvement projects

## Abstract

Background

Peripheral intravenous cannula (PIVC) insertion is a universal intervention for hospital inpatients. Previous studies have demonstrated that more than a third of inserted PIVCs remain unused in the emergency department and that there is inadequate documentation regarding the insertion and use of PIVCs. Additionally, the use of PIVC is associated with cannula-related complications. Using the PIVC care bundle attached to the guideline should help prevent cannula-related complications. As part of a quality improvement project, we aimed to perform an initial audit (1^st^ cycle), implement interventions for improvements, and then perform a re-audit (2^nd^ cycle) of our adherence with the use and completion of the care bundle for PIVC.

Methodology

An initial audit (1^st^ cycle), followed by implementation of interventions, and then a re-audit (2^nd^ cycle) of our adherence with the use and completion of the PIVC care bundle was performed. The standards/criteria used for both the 1^st^ cycle and 2^nd^ cycle of the project were obtained from our PIVC care bundle and comprised of documented evidence of the date of cannula insertion, site of cannula insertion, indication for cannula insertion, whether the cannula was inserted in a non-common site (e.g., lower limbs), cannula assessment at least every 24 hours, cannula-related complications, and whether the cannula care bundle was completed for the patient. A score of less than 75% was considered not adherent, 75-90% was partially adherent, 90-100% was adherent, and a score of 100% was considered fully adherent. The target adherence score for each standard/criterion was set a priori to 90-100%, and the results were compared between both cycles. An increase in the adherence score in the 2^nd^ cycle over the 1^st^ cycle was taken to indicate improvement, while a negative difference indicated challenges.

Results

There were 28 patients in the 1^st^ cycle and 40 patients in the 2^nd^ cycle of this project. The commonest initial indications for PIVC insertion were intravenous fluids and intravenous antibiotic administration. The hand and forearm were the commonly used sites of insertion, and none of the patients had a cannula-related complication. Compared to the 1^st^ cycle, the results of the 2^nd^ cycle demonstrated improvements in the adherence scores for all the standards/criteria, with the scores for documenting the site of insertion and using a commonly used/acceptable site indicating full adherence (100%). The score for documenting the indication for insertion indicated adherence (90-100%). The score for ensuring that the cannula was being assessed at least every 24 hours and the score for completing the care bundle both indicated partial adherence (75-90%). The score for documenting the date of cannula insertion indicated non-adherence (<75%).

Conclusions

This project has demonstrated improvement in adherence with the use and completion of the care bundle for PIVC insertion after implementing interventions for improvement. National guidance is required to produce a standard audit tool for general use. The importance of continued education, complete cannula care, and accurate documentation in enhancing adherence to consensus guidelines cannot be overemphasized.

## Introduction

Peripheral intravenous cannula (PIVC) insertion is a universal intervention for hospital inpatients. The cannula is a small flexible plastic tube inserted into a vein in the hand or arm for venous access for obtaining a blood sample for laboratory analysis and enables medication, including fluids and blood products, to be given directly into the bloodstream [1,2]. A majority of PIVCs are inserted in the emergency department when obtaining initial blood samples for laboratory analysis. However, previous studies have demonstrated that more than a third of inserted PIVCs remain unused in the emergency department and that there is inadequate documentation regarding the insertion and use of PIVCs [2].

The use of PIVC is not without complications, such as phlebitis, cellulitis, extravasation, infiltration, bleeding, hematoma, thrombotic occlusion, dislodgement, trans-fixation, bloodstream infection, and patient discomfort [3]. Additionally, there is a significant medical treatment cost that accompanies the use of PIVCs [3-6]. This invasive procedure should only be undertaken by staff who have received the training to perform this procedure. Patients who receive cannulation should have a cannula care bundle completed, the date and site of cannula insertion documented, an indication for cannula insertion documented, checked if the cannula has not been used for up to 24 hours, and also be examined daily for cannula-related complications [3]. PIVCs should be routinely changed after 72 hours of insertion to avoid cannula-related complications, such as phlebitis, cellulitis, and patient discomfort. This can only be carried out if the date of insertion is clearly documented [3,7]. Preventing cannula-related complications, particularly infections, requires good practice, starting with patient information, preparation, insertion of the cannula under aseptic conditions, cannula usage and maintenance using a care bundle, and final removal using clear guidelines [3,8].

Care bundles are a set of evidence-based, patient-focused practices or interventions (generally three to five) that aim to improve patient outcomes when done collectively and reliably. They can also be a tool to guide the delivery of a specific aspect of a patient's care, where the aim is to improve the care process and patient outcome in a structured manner or sequence, with the expectation that the impact will be greater than that of single interventions alone [8,9].

Our hospital care bundle for PIVC includes documentation of the date, time, site, and indication for PIVC insertion, who inserted the PIVC, and documentation of daily assessment, date of removal, and who removed the PIVC. Staff observation noted that the date, site, clinical indication, and daily assessment were not documented on the care bundle for every PIVC insertion.

As part of a quality improvement project, we aimed to perform an initial audit (1^st^ cycle), implement interventions for improvement, and then perform a re-audit (2^nd^ cycle) of our adherence to the use and completion of the care bundle for PIVC. Our main objectives were to improve compliance with the use and completion of the care bundle for PIVC insertion and assess for the occurrence of cannula-related complications.

## Materials and methods

This quality improvement project used the plan-do-study-act (PDSA) method [10]. The list of standards/criteria employed for both cycles was obtained from the care bundle section of our hospital guidelines. The standards/criteria included documented evidence of the date of cannula insertion, site of cannula insertion, indication for cannula insertion, whether the cannula was inserted in a non-common site (e.g., lower limbs), cannula assessment at least every 24 hours (should be removed if not being used), cannula-related complications, and whether the cannula care bundle was completed for the patient. Each patient had a "yes", "no" or "not applicable" answer to whether a standard/criterion was met. The retrieved data was entered into a Microsoft Excel spreadsheet (Microsoft Corporation, Redmond, WA, USA). Data collection and collation were carried out by two auditors over the study period. The interventions implemented between the two cycles included the presentation of the results to the medical and nursing team on the ward and intensified teaching sessions directed to doctors and nurses on the same ward. This quality improvement project was approved by and registered with our Quality Governance and Compliance Department (Project Code: 4143).

Baseline audit (1^st^ cycle)

A convenience sample of all patients who were on a particular medical ward over a five-day period in March 2024 was included in the initial prospective audit (1^st^ cycle). Data was collected prospectively over the five-day period.

Interventions

Following the initial data collection and assessment for the 1^st^ cycle, areas were identified for improvement. Interventions were designed and implemented to improve adherence with the use and completion of the PIVC care bundle. These interventions included presenting the results of the 1^st^ cycle to both the doctor and nursing teams on the ward, increasing awareness of the PIVC guidelines, provision of a copy of the care bundle section of the guidelines to staff, and individual teaching sessions regarding completion of the care bundle during PIVC insertion.

Re-audit (2^nd^ cycle)

To evaluate the impact of the implemented interventions, another set of data was collected over a similar five-day period in October 2024. A convenience sample made up of all patients who were on the same medical ward over that period were included in this 2^nd^ cycle. Data was prospectively collected as in the 1^st^ cycle.

Data analysis

The proportion of PIVCs inserted that met a standard/criterion was calculated by dividing the number of PIVCs inserted by a" yes" answer to that standard/criterion being met by the total number of applicable PIVCs inserted. The adherence score (percentage) was calculated by multiplying the derived proportion by 100. The data was presented as whole numbers and percentages. Using our in-house guide, based on national guidance, a score of less than 75% was considered not adherent, 75-90% was partially adherent, and above 90% was adherent, with 100% being fully adherent [11]. The target adherence score for each standard/criterion was set a priori to 90-100%, and the results of both cycles were compared. An increase in the adherence score (positive difference) over the 1^st^ cycle was taken as an improvement, while a negative difference indicated challenges (adherence scores less than 90% also indicated challenges).

## Results

Twenty-eight patients had a PIVC inserted in the 1^st^ cycle, while 40 patients had a PIVC inserted in the 2^nd^ cycle. The common initial indications for insertion were intravenous fluid, medications, antibiotics, insulin, and blood product administration (Table 1). The PIVCs were inserted in the commonly used/acceptable sites (hands, forearms, and antecubital fossae) in all but one patient in the 1^st^ cycle and all in the 2^nd^ cycle (Table 2). Additionally, none of the patients in the 1^st^ and 2^nd^ cycles developed any cannula-related complications.

**Table 1:** Initial clinical indications for peripheral intravenous cannula insertion in both cycles

Initial indications for cannula insertion	Number of patients	
	1^st^ cycle	2^nd^ cycle
Intravenous antibiotics	9	16
Intravenous antibiotics and fluids	6	10
Intravenous fluids	6	6
Variable-rate insulin infusion	2	4
Treatment of hypercalcemia	0	1
Blood transfusion	1	1
Other Intravenous medications	2	1

**Table 2:** Peripheral intravenous cannula insertion sites in both cycles

Cannula insertion site	Number of patients	
	1^st^ cycle	2^nd^ cycle
Right hand	3	6
Right forearm	7	12
Right antecubital fossa	2	5
Right foot	1	0
Left hand	8	7
Left forearm	5	7
Left antecubital fossa	2	3
Left foot	0	0

The adherence status for documenting the cannula site and for the use of commonly used/acceptable sites was labeled as fully adherent (100%) in the 2^nd^ cycle: the adherence score for documenting the site of insertion was similar to that in the 1^st^ cycle, while the score for the use of acceptable sites improved compared to results in the 1^st^ cycle. Adherence to documenting the indication for inserting a cannula was labeled as adherent (90-100%) in the 2^nd^ cycle, and this was an improvement compared to the result in the 1^st^ cycle. Adherence to ensuring that the cannula was being assessed at least every 24 hours and adherence to completing the care bundle were both labeled as partially adherent (75-90%) in the 2^nd^ cycle, and both of these improved compared to the 1^st^ cycle. Adherence to documenting the date of cannula insertion was labeled as non-adherent (<75%) in the 2^nd^ cycle; however, there was still marked improvement compared to the results in the 1^st^ cycle. The standards/criteria used for the 1^st^ and 2^nd^ cycles and the percentage number of patients who had each standard/criterion met in each cycle are displayed in Table 3.

**Table 3:** Results and comparison between both cycles N: total number of peripheral intravenous cannulas (PIVCs) inserted, n (%): number of PIVCs inserted that met the standard/criterion (adherence score in percentage).

Audit standards/criteria	1^st^ cycle		2^nd^ cycle		Difference in adherence (%)
	N	n (%)	N	n (%)	
Was the date of cannula insertion documented?	28	16 (57)	40	29 (72)	+15
Was the site of cannulation documented?	28	28 (100)	40	40 (100)	0
Was the indication for cannulation documented?	28	26 (93)	40	39 (97)	+4
Was the cannula assessed at least every 24 hours (and removed if not in use)?	28	19 (68)	40	33 (82)	+14
Was the cannula inserted in a commonly used /acceptable site	28	27 (96)	40	40 (100)	+4
Was the cannula care bundle completed?	28	21 (75)	40	31 (77)	+2

## Discussion

This quality improvement project, comprised of two cycles, aimed to improve our adherence to the use and completion of the PIVC care bundle section of the guidelines for the insertion of PIVC. We also wanted to assess for the occurrence of any cannula-related complications.

Improvements

The 2^nd^ cycle of the project demonstrated numerical improvement in terms of adherence scores in five of the six standards/criteria assessed in the PIVC care bundle when compared to the 1^st^ cycle. The standard regarding documentation of the site of PIVC insertion had an adherence score of 100% in both audits, so no improvement was possible. A partially adherent status to fully adherent status was assigned to five of the six standards/criteria assessed, with two of these having an adherence score of 100% as opposed to only one standard having this score in the 1^st^ cycle.

Challenges

This 2^nd^ cycle also demonstrated room for improvement (to reach an adherence score of 90% and above) in three areas, more so for areas where we were non-adherent to partially adherent. These areas included documentation of the date of cannula insertion, checking the cannula at least every 24 hours, and full completion of the PIVC care bundle. The fact that different members of staff may have been on duty during those study periods (1^st^ cycle and 2^nd^ cycle) may have been a confounding factor.

Complications of PIVC insertion

There were no cannula-related complications recorded in both cycle of this project. We noted that eight patients in the 2^nd^ cycle and four patients in the 1^st^ cycle had a PIVCs inserted in the antecubital fossae. The use of the antecubital fossae as a cannula insertion site can be uncomfortable for patients. Expert consensus does recommend the hand, followed by the wrist and then the forearm as appropriate cannula insertion sites [3].

Similar studies

Our results bear some similarities and contrasts with other reported studies shown in Table 4 [12-16]. Reduced documentation of the date of PIVC insertion and reduced documentation of the daily assessment of the PIVC for cannula-related complications and need for removal were similar themes. We did not have any cannula-related complications, which was in contrast to other studies. However, our study involved smaller patient numbers on a single ward, which makes direct comparison difficult. National guidance is required to produce a standard audit tool with guidance on adequate sample size for general use and national comparison, especially regarding cannula-related complications.

**Table 4:** A list of studies that are similar to our study

Authors	Country	Study design	Sample size	Parameters evaluated	Outcomes	Conclusions
Nelson et al., 1996 [12]	United Kingdom	Audit	100	Method of cannula attachment and dressing and cannula-related complications	Seventy-one percent of incorrectly attached cannulas developed inflammation at the insertion site	The incidence of inflammation associated with cannula insertion may be reduced by correct application of dressings and replacing the cannulas after 48 hours in situ
Bravery et al., 2006 [13]	United Kingdom	Prospective audit	625	Evaluating insertion of cannula and documentation of the insertion, the presence of phlebitis and cannula care	Poor documentation, and could not identify who inserted cannula in 37.7% of cases	Audit highlighted areas for improvement. Need for a standardized documentation for recording cannula insertion and care, and focussed education
Abolfotouh et al., 2014 [14]	Saudi Arabia	Prospective survey	842	Incidence and predictors of cannula-related complications	Cannula-related complications occurred in 39%, and predictors included female gender, insertion in fore/upper arm and infusion of medication	Better insertion techniques needed, and changing cannulas when clinically indicated rather than routinely post-72 hours
Marsh et al., 2021 [15]	Australia	Cross-sectional study of past audits	2274	Prevalence of cannula-related complications and the best sample size for calculating complications rate	Cannula-related complications rate of 7.8% to 39%	The sample size for cannula-related complications rate calculation should be 100 to 250 cannula insertions
Hoskins et al., 2022 [16]	Australia	Prospective audit	3566	Current practice related to cannula insertion, maintenance and removal, documentation, and cannula-related complications rate	Documentation lacking in respect of whether aseptic technique was used, and daily cannula site/dressing assessment was performed. Complications of blocked cannula and phlebitis occurred in 8.2% and 5%, respectively.	Low rate of complications, and recommend the use of their audit tool

Study limitations

There are a few limitations to this study. First, our study involved a small sample size compared to other reported studies. However, our study was a quality improvement project as opposed to a large audit or survey. A small sample size is usually enough to assess for change in a quality improvement project [17]. Second, this study's findings represent a single setting and limited period (five days) and may not apply to other healthcare institutions. Looking ahead, we aim to extend this study to more medical and surgical wards after implementing changes, using a more extensive audit tool, which will also include documentation of the time of PIVC insertion, date and time of removal, identity of the staff member who inserted the PIVCs, and daily assessment of the PIVCs for cannula-related complications and need for removal.

## Conclusions

We have presented a quality improvement project examining the appropriate care of peripheral intravenous cannulas (PIVC) in a busy district hospital and demonstrated improvement in adherence with the use and completion of the PIVC care bundle. We have highlighted room for improvement regarding the documentation of the date of cannula insertion and daily assessment of cannulas for removal if not used in the last 24 hours. We have also demonstrated a zero percent cannula-related complication rate, with the caveat being this was a small sample size study. The importance of continued education, complete cannula care, and accurate documentation in enhancing adherence to consensus guidelines cannot be overemphasized.
